# Protein-coding genes, long non-coding RNAs combined with microRNAs as a novel clinical multi-dimension transcriptome signature to predict prognosis in ovarian cancer

**DOI:** 10.18632/oncotarget.20457

**Published:** 2017-08-24

**Authors:** Xu Meng, Guo Jin-Cheng, Zhang Jue, Ma Quan-Fu, Yan Bin, Wu Xu-Feng

**Affiliations:** ^1^ Department of Gynecology, Maternal and Child Health Hospital of Hubei Province, Wuhan, China; ^2^ Department of Biochemistry and Molecular Biology, Shantou University Medical College, Shantou, China

**Keywords:** ovarian cancer, protein-coding genes, long non-coding RNAs, microRNAs, biomarker

## Abstract

Ovarian cancer is prevalent in women which is usually diagnosed at an advanced stage with a high mortality rate. The aim of this study is to investigate protein-coding gene, long non-coding RNA, and microRNA associated with the prognosis of patients with ovarian serous carcinoma by mining data from TCGA (The Cancer Genome Atlas) public database. The clinical data of ovarian serous carcinoma patients was downloaded from TCGA database in September, 2016. The mean age and survival time of 407 patients with ovarian serous carcinoma were 59.71 ± 11.54 years and 32.98 ± 26.66 months. Cox's proportional hazards regression analysis was conducted to analyze genes that were significantly associated with the survival of ovarian serous carcinoma patients in the training group. Using the random survival forest algorithm, Kaplan–Meier and ROC analysis, we kept prognostic genes to construct the multi-dimensional transcriptome signature with max area under ROC curve (AUC) (0.69 in the training group and 0.62 in the test group). The selected signature composed by *VAT1L*, *CALR*, LINC01456, RP11-484L8.1, MIR196A1 and MIR148A, separated the training group patients into high-risk or low-risk subgroup with significantly different survival time (median survival: 35.3 months *vs.* 64.9 months, *P* < 0.001). The signature was validated in the test group showing similar prognostic values (median survival: 41.6 months in high-risk *vs.* 57.4 months in low-risk group, *P*=0.018). Chi-square test and multivariable Cox regression analysis showed that the signature was an independent prognostic factor for patients with ovarian serous carcinoma. Finally, we validated the expression of the genes experimentally.

## INTRODUCTION

Ovarian cancer (OC) is a deadly female reproductive cancer, accounting for 5% of female cancer deaths [1]. The majority of women with ovarian cancer are always diagnosed in an advanced stage, which substantially increases the risk of early death [2, 3]. Despite advances in imaging diagnosis, preoperative and postoperative care and chemotherapy, there has been little improvement in overall survival [4–6]. Identification of clinical markers in OC is of significance to early diagnosis, select appropriate treatment and improve prognosis of patients with OC.

As the development of high-throughput sequencing technology, attempts have been made to identify molecular markers from sequencing data that affect clinical outcomes by integrating multiple profiles and clinical data [7–10]. A number of studies have shown that protein-coding genes (PCGs) involved in the many important biological processes and could be powerful predictors of tumor staging for patients in different cancers. A five-gene (*CKAP4*, *SLC40A1*, *OTOF*, *MAN2A2* and *ISPD*) signature is significantly related to patient survival in renal clear cell carcinoma patients from The Cancer Genome Atlas (TCGA) database [11]. A prognostic 7-Gene (*NHLRC3*, *ZDHHC21*, *PRR14L*, *CCBL1*, *PTPRB*, *PNPO*, and *PPIP5K2*) expression signature for stage III is constructed in colorectal cancer [12]. Recent years, long non-coding RNAs (lncRNAs) have become new players in tumorigenesis and tumor progression with an important clinical significance in prognosis due to their gene regulation function at the transcriptional, posttranscriptional and epigenetic levels [13, 14]. The well-known lncRNA named *HOTAIR* is significantly associated with breast cancer metastasis [15]. *GAS5* and *Yiya* are promising prognostic biomarkers of liver metastasis for early stage colorectal cancer patients [16]. Two immune-associated lncRNA biomarkers (RP11-284N8.3.1 and AC104699.1.1) could independently predict the survival of patients with different ovarian cancer stages [17]. Another lncRNA profile study reveals a three-lncRNA signature associated with the survival of patients with oesophageal squamous cell carcinoma [10]. Researchers have identified key lncRNAs associated with distinct stages of OC progression using a ceRNA-network driven method, and developed a ten-lncRNA signature to predict the clinical outcome of OC [18]. An eight-lncRNA signature has been found by a comprehensive analysis for lncRNA expression profile and clinical outcome of a large number of OC patients from TCGA [19]. Apart from PCGs, lncRNAs and microRNAs (miRNAs, miRs), a class of small noncoding RNAs of 18–25 nucleotides are thought to inhibit gene expression post-transcriptionally by causing mRNA degradation and/or repressing mRNA translation [20]. MiRNAs are frequently found their dysregulated expression in multiple cancers, and may function as both oncogenes and tumor suppressors [21]. *BCL11A* overexpression modulated by microRNA-30a could predict survival and relapse in non-small cell lung cancer [22]. Several prognostic and predictive microRNA markers have been identified for many types of cancers, for instance, breast cancer, hepatocellular carcinoma, lung cancer, glioma and colorectal cancer [23–29].

In summary, protein-coding genes, long non-coding RNAs and microRNAs have the prognostic potential. Therefore, the combination of PCGs, lncRNAs and microRNAs could show the clinical outcome alteration of patients with ovarian cancer more elaborately since it revealed difference in multiple transcriptome dimension. Here, we obtained the expression level of PCGs, lncRNAs and micoRNAs from a large dataset (n=407) in TCGA database and reported the first clinical multi-dimension transcriptome molecular signature which had the ability to predict prognosis in ovarian serous carcinoma patients.

## RESULTS

### Patient characteristics

All 407 patients used in this study were clinically and pathologically diagnosed with ovarian serous carcinoma. According to the International Federation of Gynecology and Obstetrics (FIGO) classification, clinical stages of the tumor were classified into stages I to IV. In our study, there were 0, 21, 321 and 62 patients in stage I, II, III and IV, respectively. Patients with missing data were not included in the study. All the other statistical information was summarized in Table 1. We divided the downloaded datasets into training and test groups randomly using the algorithm called “sample” of R program.

**Table 1:** Summary of patient demographics and clinical characteristics

Characteristic	Training set	Testing set	Total
**Age**			
Median	59	58	59
Range	38∼85	30∼87	30∼87
**Clinical stage**			
Stage I	0	0	0
Stage II	8	13	21
Stage III	163	158	321
Stage IV	31	31	62
**Vital status**			
Living	91	88	179
Dead	112	116	228

### Identification of three PCGs, three lncRNAs and three microRNAs associated with survival from the training group

We filtered out gene expression data to generate new PCGs, lncRNAs and microRNAs expression profiles (see method). Then 15426 PCGs, 8335 lncRNAs and 340 microRNAs expression values were obtained from the TCGA and TANRIC databases.

The training group (n = 203) including a relatively large patient sample size and relatively complete clinical information, were used to explore the association of survival with PCGs, lncRNAs and microRNAs. Firstly, we conducted a univariate Cox proportional hazards regression analysis of the PCGs, lncRNAs and microRNAs expression profiling data with survival time and survival status as the dependent variable, and identified a 1061-PCGs-lncRNAs-microRNAs set composed by 730 PCGs, 313 lncRNAs and 18 microRNAs which were significantly correlated with patients’ OS (P value <0.05, Figure 2A. Supplementary Table 2). Secondly, using random forest supervised classification algorithm, two PCGs, two lncRNAs and two microRNAs mostly related to the prognostic classification were selected among the 1061-PCGs-lncRNAs-microRNAs set according to the permutation important score by random survival forests-variable hunting (RSFVH) algorithm (Supplementary Figure 1).

**Figure 1:**
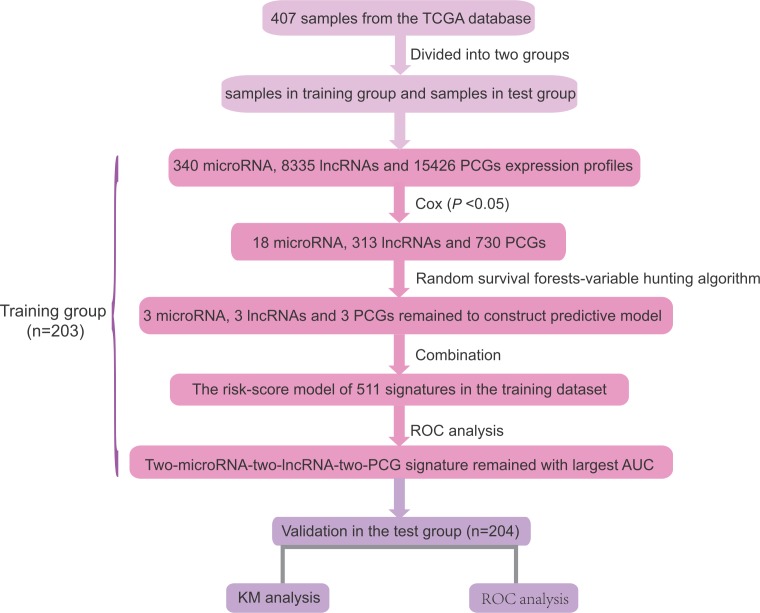
Schedule of the study The order of analyses to develop the risk score model and validate the efficiency of the signature to predict prognostic outcomes.

**Figure 2:**
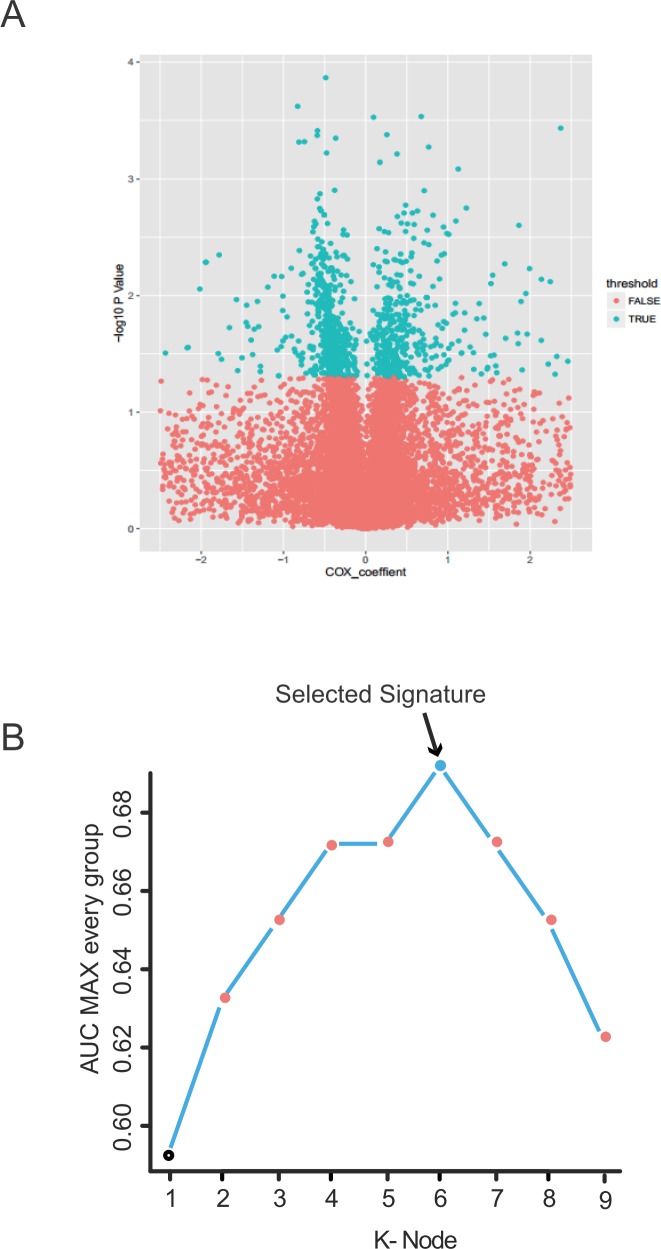
Identification of the PCGs-lncRNAs-microRNAs signature in the training dataset **(A)** Univariate Cox proportional hazards regression analysis of the PCGs, lncRNAs and microRNAs expression profiling data in the training dataset. **(B)** The procedure for identifying the final signature. The accuracies of all 511 signatures were calculated and the nine highest accuracies for k=1, 2...... 9 were shown in the plot.

### Acquisition of the prognostic PCGs-lncRNAs-microRNAs signature in the training dataset

The PCGs-lncRNAs-microRNAs set in the training dataset could have 2^9^-1=511 combination and corresponding risk score according to the signature based risk-score model (Supplementary Table 1). In order to select a better prognostic signature, we performed time-dependent ROC curve. All the risk scores of the patients were calculated as the methods described. Then the PCGs-lncRNAs-microRNAs combination composed by *VAT1L*, *CALR*, LINC01456, RP11-484L8.1, MIR196A1 and MIR148A with the max AUC was selected (Figure 2B, Table 2). The risk score of the combination composed by *VAT1L*, *CALR*, LINC01456, RP11-484L8.1, MIR196A1 and MIR148A was got as follows: Risk score = (0.47 × expression value of *VAT1L*) + (−0.52 × expression value of *CALR*) + (−0.66 × expression value of LINC01456) + (2.56 × expression value of RP11-484L8.1) + (0.14 × expression value of MIR196A1) + (−0.19 × expression value of RP11-484L8.1) + (0.14 × expression value of MIR148A). AUC of the PCGs-lncRNAs-microRNAs signature in the prognostic model was 0.69 (Figure 2B) demonstrating its good performance for survival prediction.

**Table 2:** Identities of PCGs, lncRNAs and microRNAs in the prognostic expression signature and their univariable cox association with prognosis

Ensembl ID	Gene symbol	Gene name	Coefficient^a^	*P* value ^a^	Gene expression level association with poor prognosis	Chromosome location
ENSG00000171724^b^	VAT1L	Vesicle amine transport 1 like	0.47	0.01	high	chr16:77788530-77980107:[+]
ENSG00000179218^b^	CALR	Calreticulin	−0.52	0.00	low	chr19:12938578-12944489:[+]
ENSG00000225882^b^	LINC01456		−0.66	0.05	low	chrX:17970197-18104644:[−]
ENSG00000267764^b^	RP11-484L8.1		2.56	0.01	high	chr18:48826051-48834770:[−]
hsa-miR-196a-1^c^	MIR196A1		0.14	0.03	high	chr17: 48632490-48632559 [−]
hsa-miR-148a^c^	MIR148A		−0.19	0.02	low	chr7: 25949919-25949986 [−]

a: Derived from the univariable Cox regression analysis in the training set.

b: Ensembl database

c: miRBase database

### Validation of the prediction performance of the PCGs-lncRNAs-microRNAs signature in the training dataset and the test dataset

**Figure 3:**
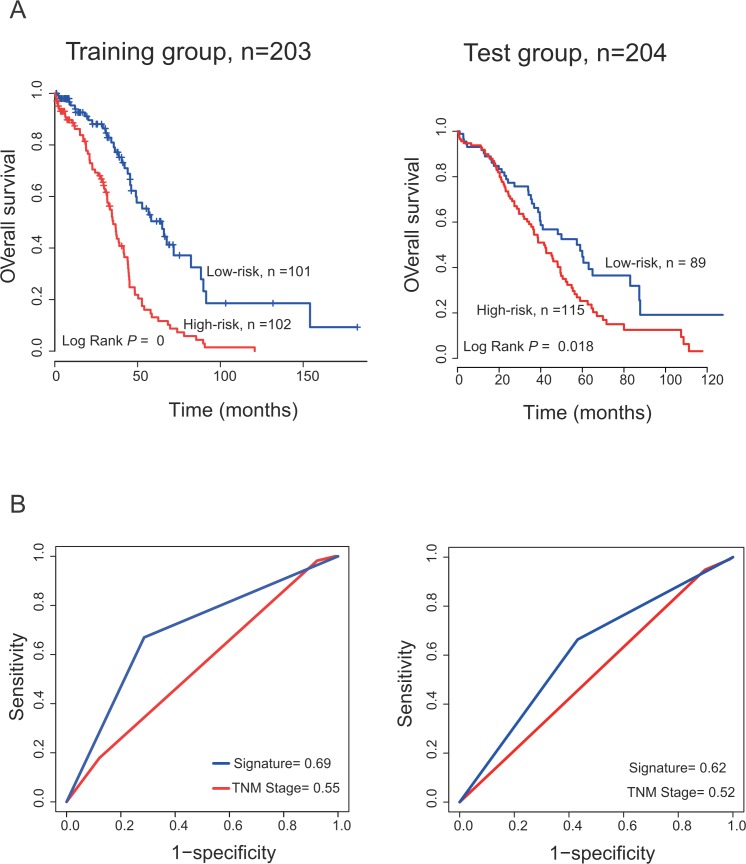
The PCGs-lncRNAs-microRNAs signature predicts overall survival of patients with OC and comparison the survival prediction power of the PCGs-lncRNAs-microRNAs signature and TNM stage **(A)** Kaplan–Meier survival curves classified patients into high- and low-risk groups using the PCGs-lncRNAs-microRNAs signature in the training and test datasets. *P* Values were calculated by log-rank test. **(B)** ROC analysis was used to compare survival prediction power between the PCGs-lncRNAs-microRNAs signature and TNM stage.

The training group patients were divided into either the high-risk group (n =102) or low-risk group (n = 101) using the median risk score as the cutoff point. Patients in the high-risk group had a significantly shorter OS than those in the low-risk group (median survival: 35.3 months *vs.* 64.9 months, log-rank test *P* < 0.001; Figure 3A, *left*). OS rates of patients in the high-risk group were less than 20% at 5 years, while more than 50% in the low-risk group.

To validate the prognostic power of the PCGs-lncRNAs-microRNAs signature for survival prediction, the constructed expression-defined PCGs-lncRNAs-microRNAs prognostic model was also evaluated in the test dataset. The test dataset patients were also classified into high-risk group and low-risk group with the same cutoff value in the training group. Kaplan–Meier curves for the high- and low-risk groups in the test dataset were shown in the *right* of Figure 3A (median survival: 41.6 months *vs.* 57.4 months, log-rank test *P* =0.018). The OS rate of patients in the high-risk group was about 33% at 5 years versus 54% in the low-risk group.

### Survival prediction performance of the PCGs-lncRNAs-microRNAs signature is independent of clinical features

To obtain a better understanding of the clinical significance of the PCGs-lncRNAs-microRNAs signature in ovarian serous carcinoma, we correlated the signature with a series of clinicopathological parameters in the combined-two groups. As shown in Table 3, there was no association between PCGs-lncRNAs-microRNAs signature and clinicopathological variables, including age and TNM stage in the training dataset.

**Table 3:** Association of the PCG-lncRNA-microRNA signature with clinicopathological characteristics in OV patients (n=203)

Variables	PCG-lncRNA signature		*P*
	Low risk *	High risk *	
**Age**			0.94
≤59	51	52	
>59	51	49	
**pTNM stage**			
unkown	1	0	0.5
II	5	3	
III	83	80	
IV	13	18	

* Low risk ≤Median of risk score, High risk >Median of risk score; The Chi-squared test; *P* value <0.05 was considered significant.

To assess whether the prognostic power of the PCGs-lncRNAs-microRNAs signature was independent of other clinical features, multivariable Cox regression analysis was performed using the signature-based risk score and other clinical features. The results of multivariable Cox regression analysis from two ovarian serous carcinoma datasets showed that the prognostic power of the PCGs-lncRNAs-microRNAs signature risk score for prediction of survival was indeed independent of these clinical features in the training group (High-risk group *vs.* Low-risk group, HR = 2.80, 95% CI 1.87–4.18, *P*< 0.001, n=203), and the same result was seen in the test group and entire dataset (Table 4).

**Table 4:** Univariable and multivariable Cox regression analysis of the PCG-lncRNA-microRNA signature and survival of OV patients in the training, test and entire group

		The training set (n=203)				The Test set (n=204)				The entire dataset (n=407)			
Variables		HR	95% CI of HR		*P*	HR	95% CI of HR		*P*	HR	95% CI of HR		*P*
			lower	upper			lower	upper			lower	upper	
**Univariable analysis**													
Age	>59 vs.≤59	1.19	0.82	1.73	0.36	0.92	0.55	1.53	0.74	1.24	0.96	1.61	0.10
pTNM stage	IV vs.II+III	1.65	1.06	2.57	0.03	1.30	0.90	1.88	0.16	1.20	0.84	1.70	0.31
PCG-lncRNA-microRNA signature	High risk vs. low risk	2.80	1.87	4.18	0.00	1.59	1.07	2.33	0.02	2.05	1.55	2.71	0.00
**Multivariable analysis**													
** Age**	>59 vs.≤59	1.24	0.85	1.81	0.43	1.48	1.01	2.16	0.04	1.36	1.05	1.77	0.02
pTNM stage	IV vs.II+III	1.49	0.95	2.33	0.21	0.97	0.67	1.38	0.86	1.16	0.87	1.55	0.30
PCG-lncRNA-microRNA signature	High risk vs. low risk	2.68	1.79	4.01	0.00	1.75	1.19	2.57	0.00	2.16	1.64	2.84	0.00

### Comparison of the survival prediction power of the PCGs-lncRNAs-microRNAs signature with TNM stage

To compare the sensitivity and specificity in survival prediction between TNM stage and the PCGs-lncRNAs-microRNAs signature, we performed ROC analysis, considering that the larger area under the ROC curve (AUC) usually implied a better model for prediction [35, 36]. In the training dataset (n=203), predictive ability of the PCGs-lncRNAs-microRNAs signature was significantly better than TNM stage (AUC Signature=0.69 *vs.* AUC TNM =0.55), which further demonstrated that the signature in our study was a novel prognostic marker with higher accuracy and had important clinical significance (Figure 3). The similar result could be seen in the test group (AUC Signature=0.62 *vs.* AUC TNM =0.52, n=204).

### Functional characterization of the selected prognostic PCGs, lncRNAs and microRNAs

**Figure 4:**
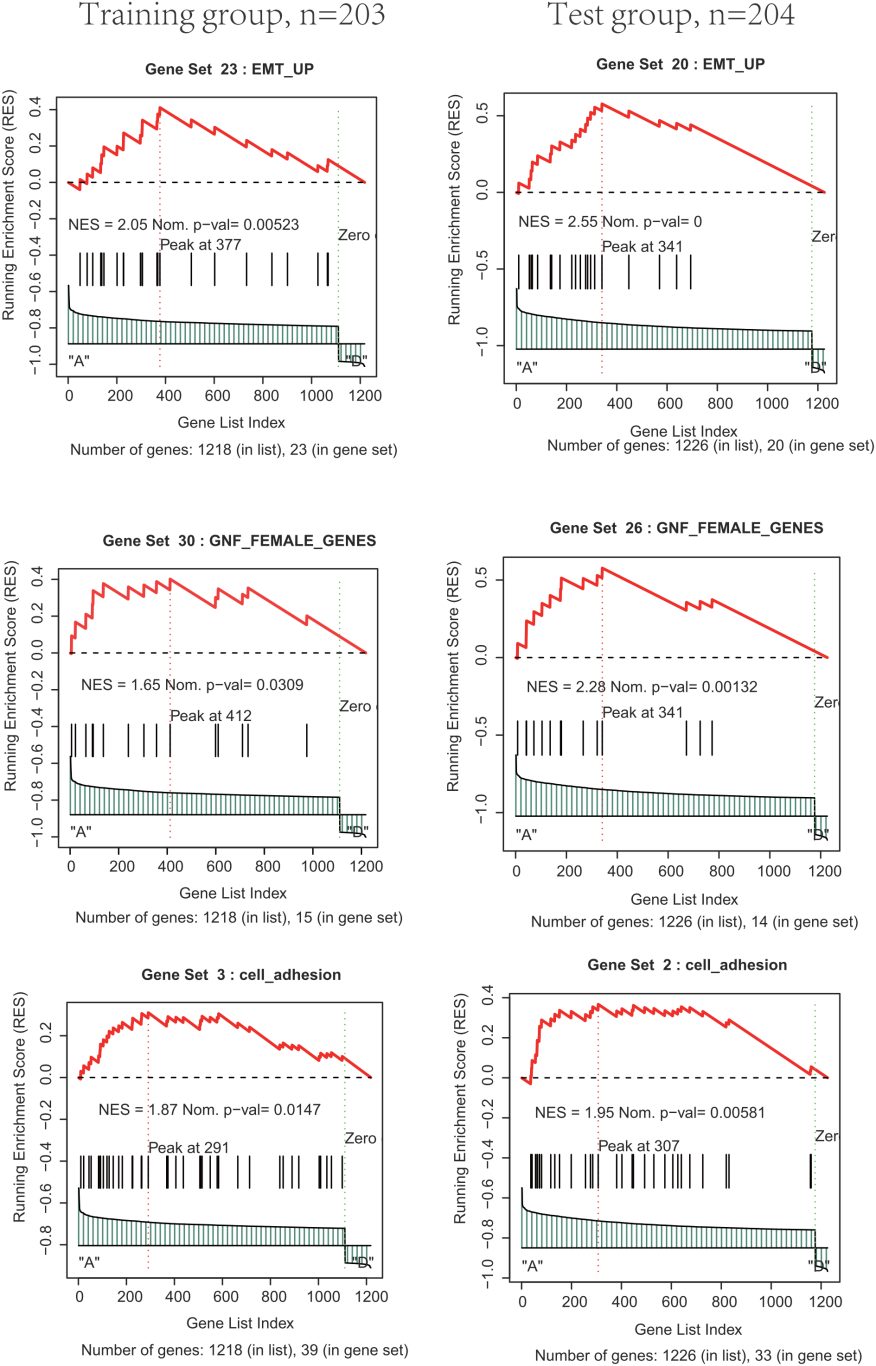
Functional enrichment of the co-expressed protein-coding genes with prognostic the two PCGs, two lncRNAs and two microRNAs by GSEA in training and test group

To further investigate the potential biological roles of this signature, the co-expressed relationships of the two PCGs, two lncRNAs and two microRNAs with those corresponding co-expressed protein-coding genes were computed using Pearson correlation coefficients in the training/test group dataset. The expression levels of 1218/1226 protein-coding genes were highly correlated with that of at least one of the selected PCGs, lncRNAs and microRNAs (Pearson correlation coefficient >0.30, *P*<0.05). GSEA analysis for these co-expressed protein-coding genes was then performed based on the whole C2 set. Several clusters of functionally related terms were observed and implied that the two PCGs, two lncRNAs and two microRNAs might be involved in tumorigenesis through interacting with those protein-coding genes that affect important biological processes such as EMT, cell adhesion, GNF FEMALE (Both in training and test groups, *P*<0.05, Figure 4).

### Validation of the PCGs, lncRNAs and microRNAs expression in an experimental cohort

**Figure 5:**
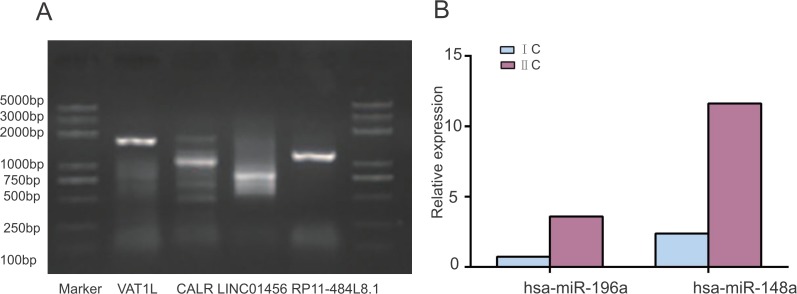
Validation of the PCGs, lncRNAs and microRNAs expression by experiment **(A–B)**.

As an additional confirmatory method of public data analysis, RNA was extracted from 2 fresh tissues of ovarian serous carcinoma patients who were operated in our hospital in September 2016 (Supplementary Table 4). The results of semi-quantified PCR for *VAT1L*, *CALR*, LINC01456 and RP11-484L8.1 were shown in Figure 5A. Using 2^−ΔΔCt^ value of the two microRNAs in each sample to evaluate the expression for MIR196A, MIR148A (Figure 5B). All six genes in the signature which was associated with the survival of OV patients by public data analysis were positive in the ovarian cancer tissues. In the future, we will enlarge the ovarian serous carcinoma samples for the real-time PCR.

## DISCUSSION

Ovarian serous carcinoma represents one of the leading causes of cancer mortality in women, exhibiting a low five-year survival rate [37]. Therefore, the identification and validation of novel biomarkers account for an important part of ovarian serous carcinoma study. On the other hand, high-throughput sequencing become more and more popular in biological studies, the complexity and multiple dimensions of these datasets make the statistical analysis difficult and challenging. Accumulating evidence suggests that PCGs, lncRNAs and microRNAs are involved in oncogenic and tumor suppressive pathways and they may serve as biomarkers [14, 38–43]. The signature composed by PCGs, lncRNAs and microRNAs, which can reflect the important biological processes from multi-dimensional levels, showed a prognostic power in OC patients.

In this study, we used different statistics and machine learning methods to identify a PCGs-lncRNAs-microRNAs expression signature that was associated with survival of ovarian serous carcinoma patients. We further revealed that the signature was an independent predictor of ovarian serous carcinoma patient survival. The multivariable Cox regression analysis was to assess the independence of the selected PCGs-lncRNAs-microRNAs signature in predicting OS. With age, pTNM stage as covariates in the regression analysis, risk score of patients based on the PCGs-lncRNAs-microRNAs signature maintained an independent correlation with OS. Taken together, these results suggested that the prognostic power of the PCGs-lncRNAs-microRNAs signature for predicting OS of OC patients was independent of other clinical features.

The expression of the six genes was validated by experiment. As for the characteristics of two PCGs, two lncRNAs and two microRNAs, the overexpression of *VAT1L*, RP11-484L8.1 and MIR196A1 was associated with shorter OS (coefficient >0) while the overexpression of the remaining *CALR*, LINC01456 and MIR148A was associated with longer OS (coefficient < 0). There is few literature about the function of the two PCGs. *VAT1L* is mainly expressed in the brain and *CALR* mutation status defined subtypes of essential thrombocythemia with substantially different clinical course and outcomes, thus it could be a potential biomarker for myeloproliterative neoplasm [44, 45]. On the another hand, miR-148a played a pivotal role in the liver by promoting the hepatospecific phenotype and suppressing the invasion of transformed cells [46], promoting cell proliferation by targeting p27 in gastric cancer cells [47], and silencing of miR-148a in cancer-associated fibroblasts resulted in WNT10B-mediated stimulation of tumor cell motility [48]. MiR-196 appears to be a vertebrate specific microRNA and it has been suggested that a rare SNP (rs11614913) that overlaps miR-196 has been found to be associated with non-small cell lung carcinoma [49, 50]. MiR-196 is correlated with metastasis and prognosis of human colorectal cancer [51], and may serve as an emerging cancer biomarker for digestive tract cancers [52]. Although the functions of these PCGs, lncRNAs, microRNAs have been inferred by bioinformatics analysis, the biological roles of the selected two PCGs and two lncRNAs in tumorigenesis are still not clear and should be investigated further.

A few of limitations in this study need to be acknowledged except limited available data about ovarian serous carcinoma. Firstly, in this study, only a fraction of human PCGs (15426 out of 30000+), lncRNAs (8335 out of 15000+) and microRNAs (340 out of 2000+) were included in the analyses. So, the prognostic PCGs, lncRNAs and microRNAs identified here might not represent all the candidates that were potentially correlated with ovarian serous carcinoma overall survival. Secondly, the specific predicted mechanisms of these PCGs, lncRNAs and microRNAs in OC need to be further study. Finally, the signature has not yet been tested prospectively in a clinical trial. Despite these drawbacks, however, the significant and consistent correlation of our PCGs-lncRNAs-microRNAs signature with overall survival in two independent datasets indicated that it was a potentially powerful prognostic marker for OC.

In conclusion, it is the first study to investigate signature composed by prognostic PCGs, lncRNAs and microRNAs in patients with ovarian serous carcinoma. The PCGs-lncRNAs-microRNAs signature can predict the survival of ovarian serous carcinoma patients with more prediction accuracy showing the signature has a bright clinical significance.

## MATERIALS AND METHODS

### Cancer PCGs, lncRNAs and microRNAs expression data in TCGA

We downloaded the microRNAs (Illumina HiSeq microRNA Seq) and mRNA (Illumina HiSeq RNA Seq V2) level 3 expression data of ovarian carcinoma from the TCGA database through the Data portal (https://genome-cancer.ucsc.edu/proj/site/hgHeatmap/). LncRNA expression datasets were obtained from the TANRIC database (http://ibl.mdanderson.org/tanric/_design/basic/index.html). All genes with missing expression values in >30% samples were removed, and then we imputed the remaining missing values by the k-nearest neighbor method. The gene expression values were log2 transformed for all subsequent analysis [30]. The selection process of the prognostic signature was shown in Figure 1.

### Construction of a weighted overall survival (OS) predictive score algorithm

We used a univariable Cox regression analysis to evaluate the relationship between the continuous expression level of each PCG or lncRNA or microRNA and patients’ OS in the training dataset. Subsequently, we developed a model for estimation of prognosis similar to what was described as follows [31, 32].

### $\text{Risk score}(\text{RS})=\sum_{i=1}^{N}\left(\text{explg }*\text{coef}\right)$

Where N was the number of prognostic PCGs, lncRNAs and microRNAs, Explg was the expression value of PCGs, lncRNAs or microRNAs, and Coef was the estimated regression coefficient of PCGs, lncRNAs or microRNAs in the univariable Cox regression analysis. This risk score model was made up of the prognostic PCGs, lncRNAs and microRNAs.

### Statistical analyses

Considering that a smaller number of PCGs, lncRNAs and microRNAs in the model would make the model more practical, we performed the random survival forests-variable hunting (RSFVH) algorithm to filter genes until two PCGs, two lncRNAs and two microRNAs were screened out [10]. The time-dependent receiver operating characteristic (ROC) curve was used to compare the sensitivity and specificity of the survival prediction of the risk score of the 511 combinations or signatures composed by the selected nine genes in the training dataset. Area under the curve (AUC) value was calculated from the ROC curve [10]. Then using the median risk score in the training dataset as a cutoff value, ovarian serous carcinoma patients in each dataset were divided into high- and low-risk groups [33]. Kaplan–Meier survival analyses were performed to test the equality for survival distributions in different groups for each ovarian serous carcinoma cohort, and statistical significance was assessed using the two-sided log-rank test. Additionally, chi-square test was used to analyze the association of the survival with the clinical attributes. And multivariable Cox regression analysis was performed to test whether the risk score was independent of other clinical features within the available data. Significance was defined as *P* < 0.05. All analyses were performed using R program (http://www.r-project.org) including packages named survival ROC, survival and random Forest SRC downloaded from Bio-conductor.

### Sample collection and preparation

The study was approved by the Hubei Maternal and Child Health Hospital, P.R. China. Fresh tissues of two ovarian serous carcinoma patients were collected, from whom underwent surgical resection (clinicopathological characteristics were listed in Supplementary Table 4). The utilization of the tissues had been through the complete patient informed consent. After being examined by a pathologist, tissues were immediately frozen in liquid nitrogen and stored at −80°C. All tumor samples contained more than 80% tumor tissue free of necrosis. All samples were coded to protect patient anonymity.

### PCR and RT-PCR experiments for validation of the gene expression

Total RNA was extracted using TRIzol (Life Technologies, USA) according to the manufacturer's protocol. cDNA was synthesized with random hexamers, PCR kit was completed by HotStar Taq Master Mix kit (QIAGEN, Germany) and real-time PCR was performed by using a QuantiTect SYBR® Green PCR kit (QIAGEN, Germany). Briefly, reverse transcription was performed according to the following conditions: 16°C, 30 min; 42°C, 40 min; 85°C, 5 min (cDNA for the RT-PCR) and 30°C, 10 min; 42°C, 60 min; 95°C, 5 min (cDNA for the PCR). RT-PCR was performed using an PAC 3000 real-time PCR system (BIO RAD, USA) according to the following conditions: 95°C, 2 min; 94°C, 10 sec; 56°C, 10 sec; 72°C, 40 sec. PCR was completed according to the criteria: 96°C, 20 sec; 52°C, 20 sec; 72°C, 5 min. Relative quantification of microRNA expression was calculated by the 2^−ΔΔCt^ method. The primer sequences of *VAT1L*, *CALR*, LINC01456, RP11-484L8.1 for PCR, and MIR196A1, MIR148A for RT-PCR were shown in Supplementary Table 3, and *ACTB* (β-actin) was used as the internal control. All were accomplished in triplicate and repeated at least three times. All methods were performed in accordance with guidelines and regulations set by the above Ethical Committee.

### Function prediction of PCGs, lncRNAs, and microRNAs by gene set enrichment analysis (GSEA)[34]

The co-expressed relationships between the prognostic PCGs, lncRNAs, microRNAs and their corresponding co-expressed PCGs were computed using Pearson correlation coefficients. Gene set enrichment analysis (GSEA) was used to predict the roles of above-obtained co-expressed PCGs (Pearson coefficient > 0.3, *P <* 0.05). Functional annotation with *P* < 0.05 was considered significant.

## SUPPLEMENTARY MATERIALS FIGURES AND TABLES







## Abbreviations

ROC: receiver operating characteristic
AUC: area under the ROC curve
CI: confidence interval
HR: hazard ratio
PCG: protein-coding gene
LncRNA: long non-coding RNA
OS: overall survival
OV: ovarian cancer
SD: standard deviation
GSEA: Gene set enrichment analysis
TCGA: The Cancer Genome Atlas
