# Supplementary material for: Protein-coding genes, long non-coding RNAs combined with microRNAs as a novel clinical multi-dimension transcriptome signature to predict prognosis in ovarian cancer
Source: Oncotarget. 2017 Aug 24;8(42):72847–59. doi: 10.18632/oncotarget.20457 (PMC5641173; doi:10.18632/oncotarget.20457)
Supplement: Supplementary file 1 [file oncotarget-08-72847-s001.pdf]

Protein-coding genes, long non-coding RNAs combined with microRNAs as a novel clinical multi-dimension transcriptome signature to predict prognosis in ovarian cancer

SUPPLEMENTARY MATERIALS

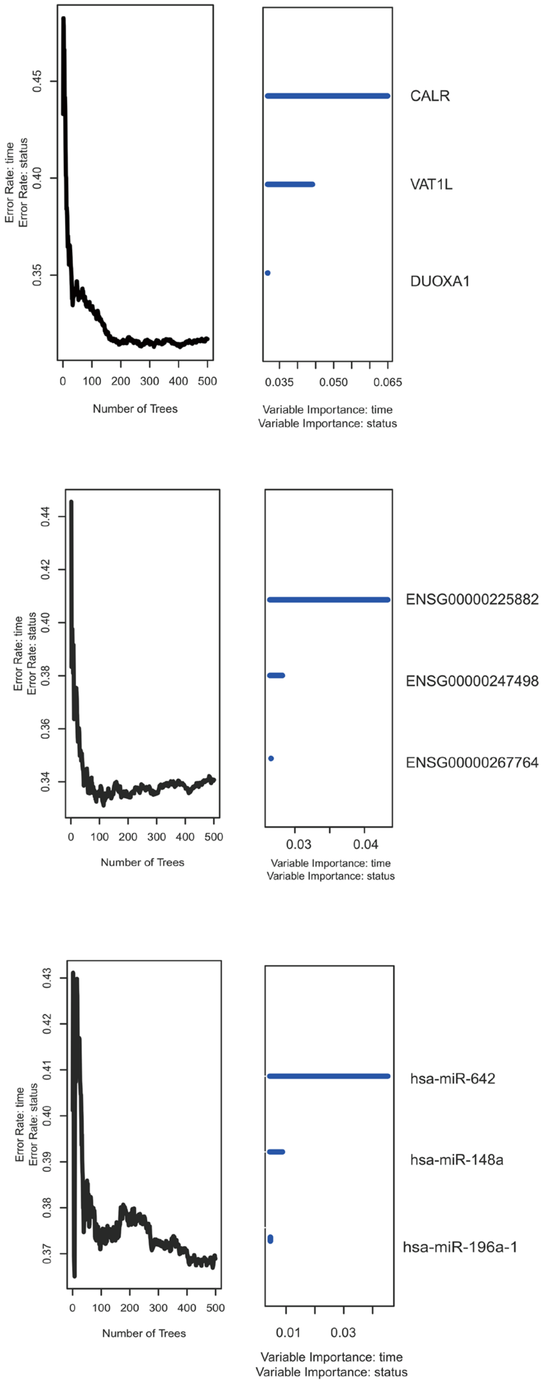

Supplementary Figure 1: Random survival forests-variable hunting analysis reveals the error rate for the data as a function of trees.

Supplementary Table 1: The signature composed of PCGs, lncRNAs and microRNAs in the training and test datasets (n=203)

See Supplementary File 1

Supplementary Table 2: PCGs, lncRNAs and microRNAs of Univariate Cox regression analysis (P <0.05) in the training set (n=203)

See Supplementary File 2

Supplementary Table 3: Primer sequences for PCR and real-time RT-PCR

Gene	Forward (5' to 3')	Reverse (5' to 3')
VAT1L-CDS	CGACCCTAACTTGGGACTAT	GACAAACACGACACGCACT
CALR-CDS	AACTACAAGGGCAAGAACGT	AGGGCTGAAGGAGAATCAAAGA
LINC01456	AAGCAAGCCTGGGCAATG	TAACAAATCACCCTGAAAC
RP11-484L8.1	AGGGAGTTCCTCTAAAGCG	ATCCACTGACCAGCCAAA
hsa-miR-196a	AGGTAGTTTCATGTTGTTGG	GTCGTATCCAGTGCGTGTCGTGGAGTCGG CAATTGCACTGGATACGACCCCAAC
hsa-miR-148a	TCAGTGCACTACAGAACTTT	GTCGTATCCAGTGCGTGTCGTGGAGTCGG CAATTGCACTGGATACGACACAAAG
ACTB	AGCGAGCATCCCCCAAAGTT	GGGCACGAAGGCTCATCATT

Supplementary Table 4: Patients' clinical information

NO.	Age	CA125(U/ml)	Grade	Clinical stage	Ascites(ml)
1	66	359.9	high	IC	100
2	45	94.7	low	IIC	0
